# Genomic consequences of selection and genome-wide association mapping in soybean

**DOI:** 10.1186/s12864-015-1872-y

**Published:** 2015-09-03

**Authors:** Zixiang Wen, John F. Boyse, Qijian Song, Perry B. Cregan, Dechun Wang

**Affiliations:** Department of Plant, Soil and Microbial Sciences, Michigan State University, 1066 Bogue St., Rm. A384-E, East Lansing, MI 48824-1325 USA; Soybean Genomics and Improvement Laboratory, Agricultural Research Service, United States Department of Agriculture, Beltsville, MD 20705 USA

**Keywords:** Selective sweep, Single nucleotide polymorphisms, GWAS, Soybean

## Abstract

**Background:**

Crop improvement always involves selection of specific alleles at genes controlling traits of agronomic importance, likely resulting in detectable signatures of selection within the genome of modern soybean (*Glycine max* L. Merr.). The identification of these signatures of selection is meaningful from the perspective of evolutionary biology and for uncovering the genetic architecture of agronomic traits.

**Results:**

To this end, two populations of soybean, consisting of 342 landraces and 1062 improved lines, were genotyped with the SoySNP50K Illumina BeadChip containing 52,041 single nucleotide polymorphisms (SNPs), and systematically phenotyped for 9 agronomic traits. A cross-population composite likelihood ratio (XP-CLR) method was used to screen the signals of selective sweeps. A total of 125 candidate selection regions were identified, many of which harbored genes potentially involved in crop improvement. To further investigate whether these candidate regions were in fact enriched for genes affected by selection, genome-wide association studies (GWAS) were conducted on 7 selection traits targeted in soybean breeding (grain yield, plant height, lodging, maturity date, seed coat color, seed protein and oil content) and 2 non-selection traits (pubescence and flower color). Major genomic regions associated with selection traits overlapped with candidate selection regions, whereas no overlap of this kind occurred for the non-selection traits, suggesting that the selection sweeps identified are associated with traits of agronomic importance. Multiple novel loci and refined map locations of known loci related to these traits were also identified.

**Conclusions:**

These findings illustrate that comparative genomic analyses, especially when combined with GWAS, are a promising approach to dissect the genetic architecture of complex traits.

**Electronic supplementary material:**

The online version of this article (doi:10.1186/s12864-015-1872-y) contains supplementary material, which is available to authorized users.

## Background

The cultivated soybean, *Glycine max* (L.) Merr., was domesticated in China from its wild ancestor, *G. soja* Sieb. et Zucc., which has a wide geographic distribution in East Asia. Although the exact series of steps by which soybean was domesticated is still unknown, the divergence between *G. max* and *G. soja* likely happened ~0.8 million years ago based on inter-genomic comparison analysis [1]. The long time period since divergence and probably multiple domestication events resulted in a multitude of localized *Glycine max* landraces [2], which are adapted to different environments. Currently*,* there are 45,000 accessions of *G. max* in *ex situ* collections around the world [3]. Subsequent to domestication, soybean has been subject to intensive improvement efforts over the past century. Despite the seemingly vast reservoir of genetic diversity in *G. max*, just 346 (0.77 %) of those landraces account for 76.29 % of the nuclear contribution of 1300 Chinese soybean cultivars released between 1923 and 2005 based on pedigree analysis [4]. Major modern U.S. soybean varieties released between 1947 and 1988 can be traced back to only 80 accessions from a small area in northeastern China. Approximately 86 % of the collective parentage was contributed by just 17 of the 80 landraces [5]. These landraces provided the genetic material for modern breeders to develop varieties by enhancing traits controlling agricultural productivity and performance, such as high yield, reduced branching and resistance to biotic and abiotic stress. Consequently, the genome of soybean varieties might have experienced strong selection at genes controlling these traits during domestication and subsequent genetic improvement.

How can one detect this selected class of genes that contributes to the variation of agronomic traits? Historically, quantitative trait locus (QTL) mapping has been used to localize genomic regions underlying phenotypic variation. Since only small numbers of recombination events can be accumulated over the few generations during the development of a recombinant inbred line mapping population, this approach has rarely led to candidate gene isolation [6]. Association mapping, which exploits historical recombination events, has become a powerful alternative to linkage mapping for the dissection of complex trait variation at the sequence level [7–9]. In soybean, genome-wide association study (GWAS) has been used to dissect various traits, such as disease resistance, yield and quality related traits [10–12].

Apart from the above mentioned QTL and association analysis methods, selective sweep analysis is another approach that can be used to detect loci of potential agronomic importance. A selective sweep alters the allele frequencies of single nucleotide polymorphisms (SNPs) in the vicinity of the selected allele, and causes (i) reduced local variability, (ii) a distorted pattern of genetic variation, (iii) increased linkage disequilibrium (LD) and (iv) extended haplotype structure [13, 14]. These characteristics can be used to scan a genome for genes involved in recent adaptation. Recently, a cross-population composite likelihood ratio (XP-CLR) method [13] was used to scan for extreme allele frequency differentiation during domestication and improvement in maize. Approximately, 7.6 % of the maize genome showed multiple signatures of selection and 3040 genes were found to be involved in improvement [15]. In the case of soybean, multiple studies have focused on contrasts of local variability and different patterns of LD among elite soybean cultivars, landraces and wild relative, *G. soja*. [16–19]. Recent inter-genomic comparisons among the genome sequences of 8 *G. soja* and *G. max* accessions identified 682 genes showing signatures of positive selection including some lineage-specific genes and genes with copy number variation [1]. However, most of these studies had limitations either in shallow sampling [1, 16, 19], weak power of statistical methods [17] or limited genome coverage [18]. Many selection signals may, therefore, have remained un-detected.

In this study, a high-density customized oligonucleotide array (52,041 SNPs) was used to genotype 342 traditional landraces and 1062 improved soybean lines. On the basis of quantified variation in nucleotide diversity, linkage disequilibrium and population structure, XP-CLR statistics [13] were used to identify the regions of the genome most affected by selection for traits targeted by breeding. Combined with 9 agronomic traits data collected from multiple environments, a substantial number of loci potentially underlying these traits were identified by GWAS. Specifically, we aimed to determine (*i*) the extent to which the genetic diversity throughout the genome has been impacted by selection, (*ii*) the regions of the genome that have been affected by selection during soybean improvement, and (*iii*) whether our candidate regions are truly enriched for genes affected by selection of traits targeted by breeding.

## Results and discussion

### Effect of selection on diversity and linkage disequilibrium

**Fig. 1:**
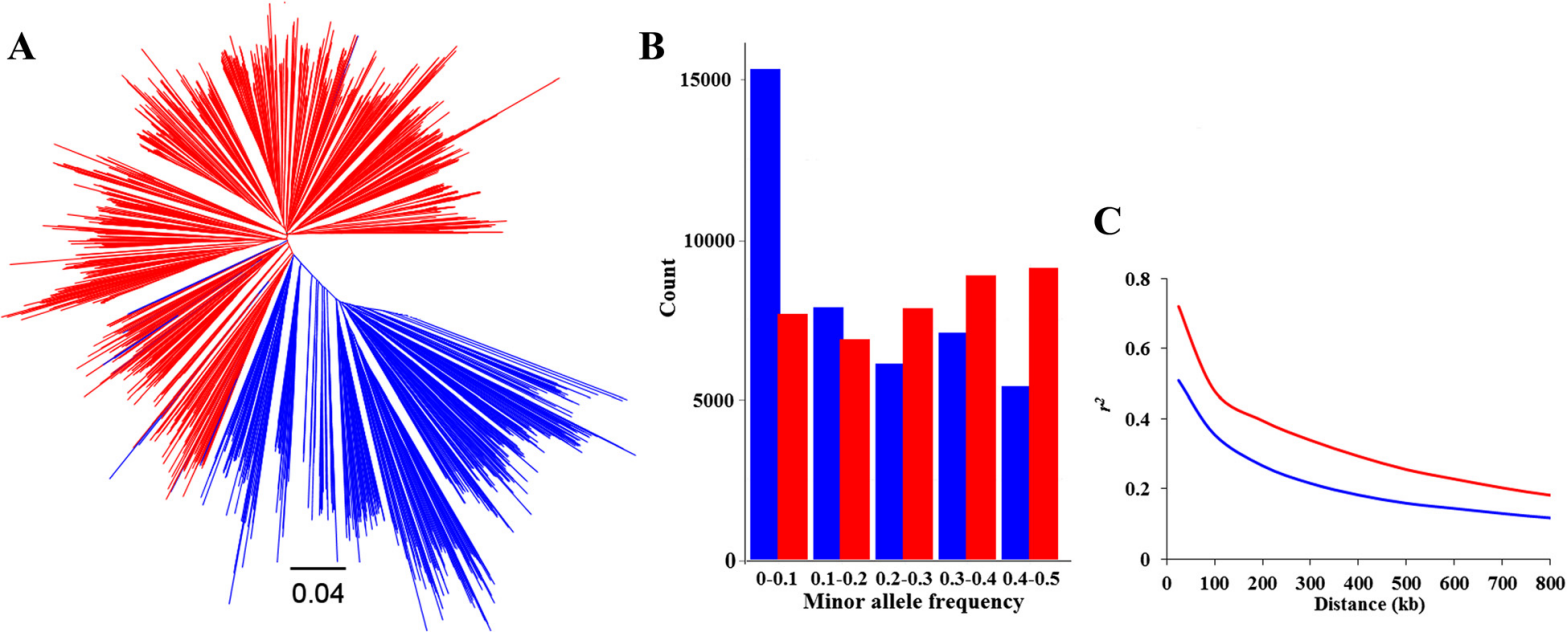
Genetic divergence of soybean landraces and improved lines. **a** NJ tree of all soybean accessions tested in this study. Accessions in the neighbor-joining tree are represented by different colors: landraces (blue) and improved lines (red). **b** Comparison of minor allele frequencies (MAF) between landraces (blue) and improved lines (red). **c** Genome-wide average LD decay estimated from landraces (blue) and improved lines (red)

To better understand the patterns of genomic modification imposed by selection, profiles of 52,041 SNPs were characterized in 342 soybean landraces and 1062 improved lines. After quality control, a high-density haplotype map, comprised of genotypes for 35,708 SNPs, was generated for all sampled accessions. Phylogenetic relationships among these accessions were determined using the genetic distances calculated from these SNPs. The resulting neighbor-joining (NJ) tree showed two divergent groups belonging to the landraces and improved lines, except for a few admixed genotypes between the two groups (Fig. 1a). This result raises the possibility that a stronger genome-wide bottleneck has occurred as a result of improvement resulting from soybean breeding. To evaluate the degree to which genetic diversity throughout the genome has been impacted by selection, we further quantified variation in nucleotide diversity, linkage disequilibrium (LD) and haplotype block structure for the two populations.

Although our estimates of genetic diversity in improved lines may be inflated by the larger sample size, the soybean landraces were still more diverse than the improved lines in terms of both genetic richness (*A*_*i*_) and diversity index (*H*_*i*_) (Table 1). A significant reduction of genetic diversity was observed across every chromosome (Table 2). Note that the improved lines retained 70 % (*H*_*i*_) of the diversity present in the landraces. This is not only close to previous observations [3], but also close to the 77 % diversity that maize elite inbred lines retained across 21 loci relative to the diversity found in maize landraces [20]. An examination of allele frequency distributions at all polymorphic loci showed that landraces contained a larger number of rare SNPs (MAF <0.1) than improved lines. Additionally, 50 % of the rare SNPs in the landraces were not present in the improved lines (Fig. 1 b). Several factors could be responsible for the genetic erosion in improved lines relative to that found in the landraces. One factor is that modern plant breeding usually introduces intensive selection within a narrow range of landraces with limited allele introgressions over time. The other factor might be that selection would probably reduce the diversity and changes allele frequencies in the DNA surrounding the loci that are targets of selection; the following selective sweep analysis and GWAS confirmed this point.

**Table 1:** Characteristics of SNPs tested in soybean landraces and improved lines

Population	MAF^b^		Genetic diversity		Haplotype blocks		Extent of LD decay (kb)	
	>0.01	>0.05	*A*_i_	*H*_i_	No.	Size (kb)	Max.	Average
Landraces (342^a^)	38,453	35,708	81,034	0.33	5513	105	427	187.8
Improved lines (1062)	35,189	30,651	72,226	0.23	3180	245	430	233.6

^a^No. of accessions, ^b^MAF, minor allele frequency

**Table 2:** Summary of genetic diversity, LD decay and selective sweeps across 20 chromosomes within the two soybean populations

	Genetic diversity index		LD decay rate (kb)		Selective sweeps	
Chr.	Landraces	Improved lines	Landraces	Improved lines	Feature No^a^.	XP-CLR^b^
1	0.33	0.27	150	226	4(10)	10.71
2	0.37	0.27	198	276	13(56)	14.56
3	0.35	0.24	100	135	10(28)	14.81
4	0.34	0.18	158	113	15(59)	12.55
5	0.31	0.26	177	270	4(14)	11.82
6	0.35	0.21	106	206	6(32)	15.22
7	0.33	0.23	276	235	8(13)	10.47
8	0.33	0.22	172	242	6(25)	13.05
9	0.38	0.27	172	190	4(22)	14.11
10	0.32	0.25	126	158	5(17)	15.57
11	0.25	0.18	197	176	3(13)	10.72
12	0.29	0.15	160	175	5(11)	10.98
13	0.35	0.26	115	311	5(19)	11.94
14	0.34	0.27	224	317	4(22)	13.03
15	0.36	0.27	295	305	7(16)	12.43
16	0.34	0.28	110	101	4(10)	15.53
17	0.34	0.24	106	171	6(27)	14.7
18	0.33	0.32	427	375	4(17)	13.45
19	0.34	0.15	314	430	5(21)	12.18
20	0.32	0.19	172	259	6(41)	12.93

^a^The numbers in brackets indicate the number of 20 kb-windows that exceeded the 1 % genome-wide cutoff threshold value. ^b^XP-CLR indicates the cross-population composite likelihood ratio value

Since increased LD is another hallmark of genetic bottlenecks, we compared the haplotype block size and LD decay rate of the soybean landraces with that of the improved lines. In general, both landraces and improved lines exhibited low LD decay rates (Fig. 1c). In comparison, the extent of LD decay increased from 187.8 kb for landraces to 233.6 kb for improved lines. Additionally, the average haplotype block size in the improved lines (245 kb) was more than twice that in the landraces (105 kb) (Table 1). The increase of the extent of LD decay and block size in improved lines may be caused by the increased hitchhiking of deleterious mutations and loss of genetic diversity during the soybean improvement [21]. These LD decay estimates are smaller than previously published values in landraces of 500 kb [11] and in improved lines of 270 kb [10]. This difference may be attributed to low genome coverage of markers and fewer genotypes in previous studies. Because soybean is a self-pollinated species, we expect a greater extent of LD than in out-crossing species [22]. The extent of LD in soybean is similar to that of the self-pollinated species rice (∼123-167 kb) and sorghum (∼150 kb) [23, 24] but much greater than in maize (1-10 kb), an out-crossing species [25].

Given that our average inter-marker distance (density) is 35 kb, we expect to have reasonable power to identify common variants of large effect associated with agronomic traits in association mapping. However, the low rate of LD decay in soybean also may lead to resolution limitations for the association mapping.

### Profile of genetic differentiation and population structure

**Fig. 2:**
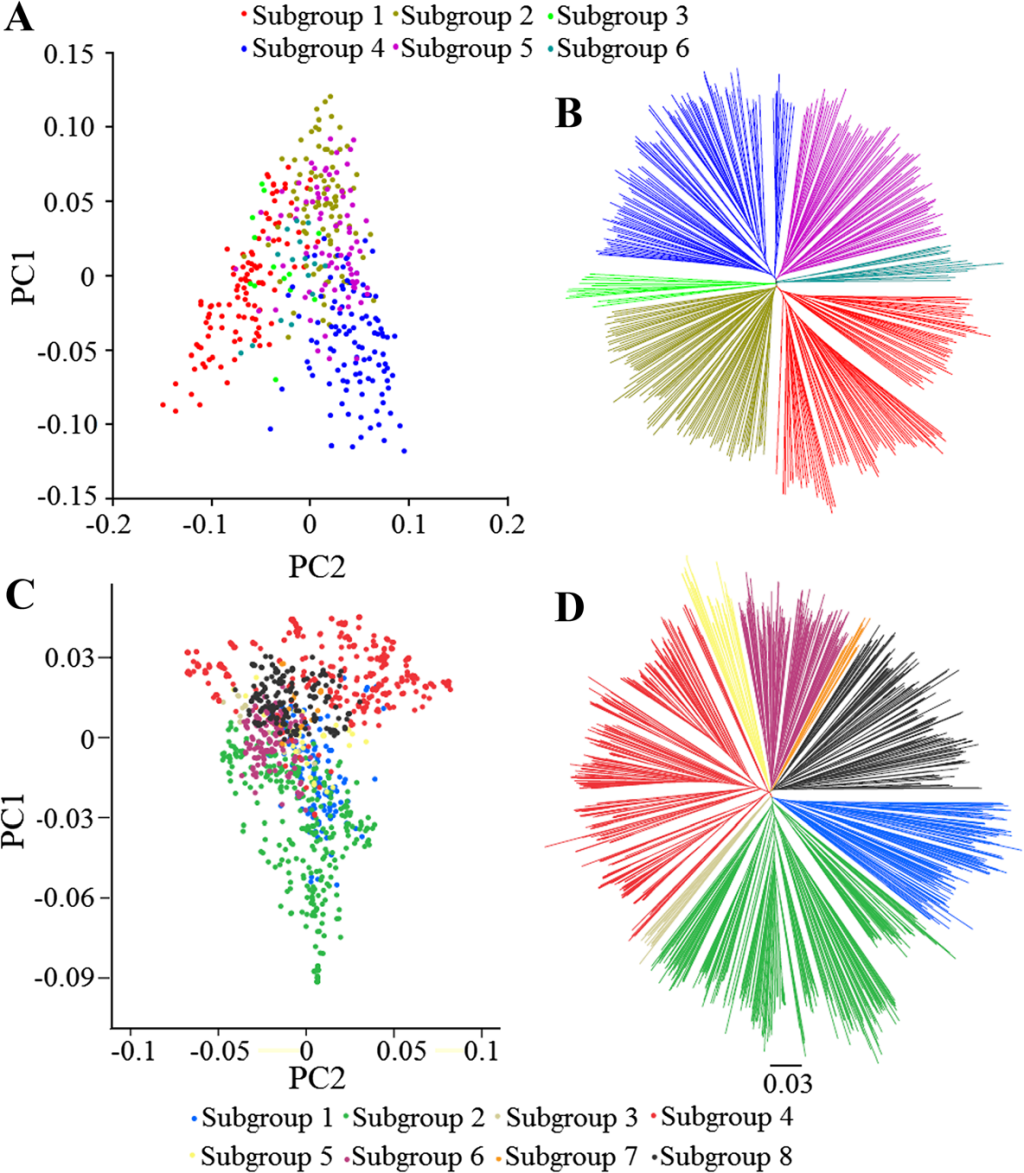
Population structures of soybean landraces and improved lines. **a** PCA plots of the first two components of 342 accessions of soybean landraces. **b** NJ tree of soybean landraces. The 6 subgroups identified from the tree are color-coded in **a** and **b**. **c** PCA plots of the first two components of 1062 accessions of improved lines. **d** NJ tree of improved lines. The 8 subgroups identified from the NJ tree are color-coded in **c** and **d**

To understand the geographic structure of genetic diversity and population stratification, NJ tree plots and principal components analysis (PCA) were applied to determine the relatedness among the sampled accessions. The resulting NJ trees and PCA plots showed that the landraces had 6 subgroups, whereas the improved lines had 8 (Fig. 2). The measure of population differentiation, *F*_ST_, averaged 0.139 among the subgroups of improved lines (Additional file 1: Table S1). This is close to that between different rice populations (*F*_ST_ = 0.14) [23]. The *F*_ST_ among the subgroups of landraces was estimated at 0.10 on average (Additional file 1). This estimate is slightly less than that of the improved lines as well as previously published values [18], and close to that between different human populations (*F*_ST_ = 0.12) [26].

Based on analysis of origin of each accession for every subgroup, we found that overall population structure was based upon geographic origin and maturity group for the landraces and improved lines, respectively (Fig. 2 and Additional file 2). The Chi-square test was used to test whether the 6 SNP-data-based subgroups were associated with geographic origin in the landraces (Additional file 2). The results showed very significant association (*p* < 0.0001) between the two grouping factors. Furthermore, significant associations (*p* < 0.0001) were also observed between the 8 SNP-data-based subgroups and maturity groups in the improved lines (Additional file 2). Thus, we speculate that photoperiodic response may have been at least as important as geographic isolation in shaping genetic differentiation of soybean. Taken together, these results highlight the need to account for population structure when performing association analyses in soybean.

### Genome wide selective sweep analysis

**Fig. 3:**
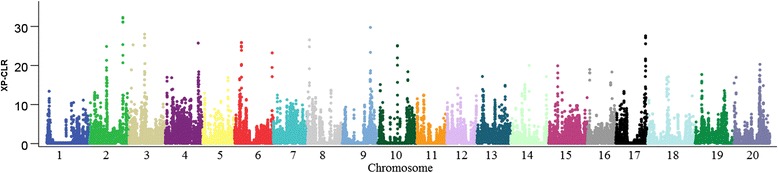
Genome-wide visualization of selection during soybean improvement. Each dot represents a non-overlapping window of 20 kb with cross-population composite likelihood ratio (XP-CLR) values indicated along the *y* axis and physical position indicated along the *x* axis

Artificial selection has probably left detectable signatures within the genome of elite soybean cultivars. In order to identify regions of the genome most affected by artificial selection during improvement, signals of selective sweeps were screened by a XP-CLR approach [13] to compare the improved lines versus the reference panel of landraces (Fig. 3).

Of the 52,041 total SNPs, 69 % of SNPs were polymorphic with MAF >0.05. These filtered SNPs were used in the genome scan, resulting in coverage of 88 % of 20-kb-windows. Using a threshold by which the top 1 % of XP-CLR values (9.49) were selected, a total of 472 20 kb-windows exceeded the cutoff value. Adjacent windows within the same LD block were grouped into ‘features’, with each likely representing the effect of a single selective sweep. After the joining of adjacent 20 kb windows, 125 features were identified (Additional file 3). Mean and median feature width are 75.2 and 60 kb, respectively, with approximately 1.0 % of the genome contained in candidate features. These features showed little overlap with previously identified regions that were found to be impacted by improvement [17, 19], indicating that ascertainment biases (caused by SNP localization), statistical methods and reference sample differences may have influenced identification. When we replaced our reference panel with the same panel of 96 landraces used by Song et al [17] and calculated the corresponding XP-CLR (data not shown) again. The overlapping proportion of selective sweep before and after replacement was 68 %. So we conjecture that the statistics method is the key factor causing differences in identification of selection sweeps. Meanwhile, since major proportion of selective sweep can be reproducibly identified with different reference panel, we are confident about the reproducibility of our findings.

Additionally, high correlation between XP-CLR scores and the width of candidate features was found (R^2^ = 0.46, *p* < 0.01). The correlation between feature width and XP-CLR is larger than that found in improvement scans in maize (R^2^ = 0.008, *p* = 0.0187). The reason for this may be attributed to the observation that LD is more extensive in self-pollinated species than in cross-pollinated species. Moreover, average significant XP-CLR values (13.3) from our improvement scan were substantially lower than those observed for maize improvement (XP-CLR = 19.1) [15]. One possible explanation for this may be that soybean has been subject to less intensive breeding efforts than maize. Note that different chromosomes showed different levels of selective sweeps with large variation of feature number and size among different chromosomes (Table 2). Among the 20 chromosomes, Chromosome 4 has the largest number of selective sweep features, which suggests that this chromosome might be the one most affected during soybean improvement (Table 2). Other studies have demonstrated that Chromosome 4 is rich in QTLs for seed size [27], yield components [28] and disease resistance [29].

We assigned the gene closest to the 20-kb window with the maximum XP-CLR score in each LD block as the most likely candidate (Additional file 3). These candidates can be classified into different gene categories, with a significant higher proportion (using Fisher’s exact test relative to whole genome level) of genes associated with DNA (or RNA) binding and catalytic processes (Additional file 3). About 40 % of these candidates (or orthologous genes) have been anticipated to be involved in improvement of soybean or maize (Additional file 3) [1, 15]. Several candidates are interesting based on their homology, even though their exact functions in soybean are not known. For instance, *Glyma.14 g201100* has protein homology to *OsNAC9*, which affects grain yield and drought resistance in rice [30]; *Glyma.02 g138100*, highly expressed in soybean seed, is orthologous to maize SAD that is involved in converting stearic acid to oleic acid [31]. A few genes among our improvement candidates have been functionally characterized in soybean. For example, *Glyma.18 g022400* (an amino acid transmembrane transporter) is one of the three genes known to contribute to SCN resistance [32]; *Glyma.04 g101500* (GmCRY1) is a major regulator of photoperiodic response in soybean and correlates with latitudinal distribution of soybean [33]; *Glyma.08 g109300* (a chalcone synthase gene) is one of the genes which controls the distribution of seed-coat color by inhibiting coloration over the entire seed coat [34]. The three genes showed strong selection signals that may be caused by positive selection for resistance to SCN, wider geographical regions of adaptation and yellow seed coat color, respectively, by which the favorable alleles have been captured and accumulated in this set of improved lines.

On one hand, the XP-CLR method we used is much more robust to ascertainment bias in SNP discovery than methods based on the allele frequency spectrum, and more powerful than the CLR-test and Tajima’s *D* test [13]. These advantages increase our power to detect selection and provide important insight into the pathways and genes responsible for soybean improvement. On the other hand, we recognize at least two limitations in our analysis. First, our SNP coverage may be still insufficient to capture all variants that lead to a conservative test for selection. As the costs of genotyping become reasonably low, additional studies with higher re-sequencing depth will be helpful for identifying new candidate genes related to soybean improvement. Second, rather than being the direct targets of selection, some regions could have hitchhiked along with another target of selection because of the nature of high level of LD in soybean. It is thus possible that some of our candidate genes could be false positives.

### Validating putative sweeps with GWAS

Although the candidate regions, which most likely experienced a selective sweep, have been identified, the functions or the phenotypes associated with most of the genes in these regions remain elusive. If our candidate regions are truly enriched for genes affected by selection of breeding target traits (such as yield, protein and oil content), they should at least partially overlap with QTL regions associated with these traits, whereas no overlap of this kind should be observed for non-selection-target traits (such as pubescence and flower color). To validate this hypothesis and dissect genetic architecture of agronomic traits, GWAS was performed on 9 agronomic traits. To increase the reliability of agronomic trait data, especially for quantitative traits, phenotyping was conducted at 7 different locations over a 6-year period. With the exception of lodging, we observed abundant phenotypic variation and normal distribution for grain yield, plant height, maturity date and protein and oil content in the tested accessions (Additional files 4 and 5). As for the 3 quality traits, seed coat color, pubescence and flower color, significant distorted distribution was observed only for seed coat color (Additional file 5).

### Overall profile of GWAS results

Two statistical models were used in our GWAS. As shown in the quantile-quantile (QQ) plots (Additional file 6), the distribution of observed -log10 *P*-values from the general linear model (GLM), which did not include population structure (Q) and familial relatedness (K), departed from the expected distribution under a model of no association with significant inflation of nominal *P*-values. The mixed linear model (MLM) model that includes Q and K allowed us to compress the excess of low *P*-values for these traits (Additional file 7). Lower inflation of nominal *P*-values was consistently observed when the MLM model was used but not when the simple model was used. Therefore, only the results from the analysis with the MLM model are presented below.

**Fig. 4:**
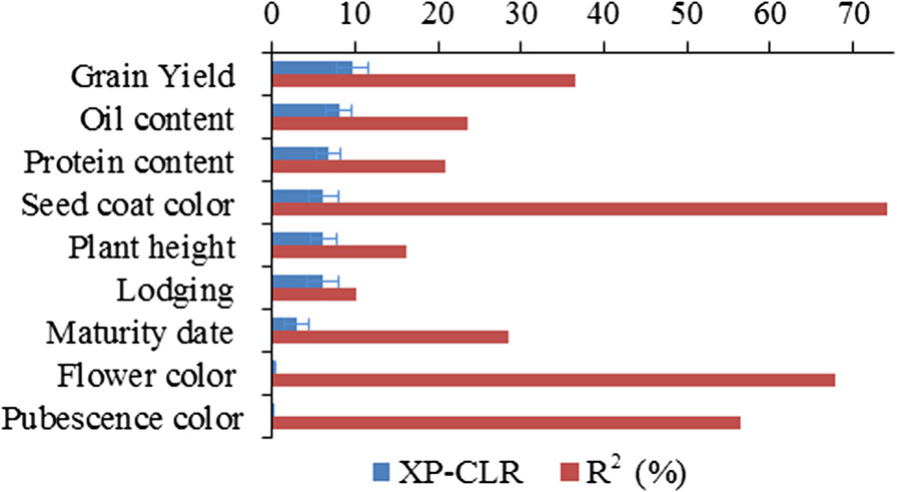
Contributions of identified loci to phenotypic variance (R^2^) of 9 traits and the corresponding XP-CLR value

A total of 417 SNPs were significantly associated with 9 agronomic traits. The results of significant SNPs discovered in two populations are summarized in Additional file 8 and Table 3. We successfully identified known associations (genes or QTLs previously reported in soybean), as well as new candidate loci in the genome for the 9 traits. The identified loci explained an average of 37.1 % of the phenotypic variance. Corresponding XP-CLR values for those loci were investigated one by one (Fig. 4 and Additional file 8).

**Table 3:** A subset of SNPs significantly associated with 9 agronomic traits

Trait	Chr.^a^	Position^b^	MAF	Allele	*P* value	R^2^(%)	XP-CLR	Known loci^c^
Grain yield	1	55794390	0.06	T/C	2.38 × 10^-6^	2.6	1.60	
	3	47136179	0.48	T/C	2.69 × 10^-5^	2.4	0.68	15-4
	4	43653965	0.10	T/C	1.59 × 10^-6^	2.7	18.20^d^	12-2
	8	39270868	0.21	T/C	5.50 × 10^-8^	4.5	4.25^e^	
	14	46260055	0.07	A/G	3.80 × 10^-7^	3.2	17.01^d^	3-4
Protein content	7	7058915	0.11	T/C	1.46 × 10^-7^	3.2	3.55	24-4
	9	1195313	0.47	T/C	4.57 × 10^-7^	3.0	4.76^e^	
	10	47656484	0.14	T/C	2.88 × 10^-5^	2.0	9.02^e^	5-4
	15	5312718	0.20	A/G	1.49 × 10^-5^	2.3	1.94	5-4
	20	36078120	0.06	T/G	1.62 × 10^-5^	2.1	7.63^e^	26-5
Oil content	1	52863692	0.30	A/G	6.66 × 10^-6^	2.5	8.45^e^	24-21
	6	14511997	0.08	A/G	1.23 × 10^-5^	2.4	8.93^e^	36-2
	10	6572950	0.08	A/G	2.29 × 10^-5^	2.6	10.37^d^	
	11	1859395	0.06	T/C	7.63 × 10^-6^	2.3	2.10	
	14	5340642	0.16	A/G	7.23 × 10^-7^	2.9	4.14	2-6
Lodging	2	7785541	0.19	T/C	3.81 × 10^-5^	2.6	7.90^e^	7-1
	10	44437412	0.13	A/C	4.96 × 10^-6^	2.3	9.02^e^	
	10	44723907	0.12	A/G	5.06 × 10^-7^	2.8	7.35^e^	20-6
	10	44747924	0.12	A/G	1.85 × 10^-6^	2.6	7.35^e^	20-6
	19	47672198	0.39	T/C	8.14 × 10^-6^	2.4	0.227	4-3
Plant height	8	10789902	0.18	T/C	6.84 × 10^-6^	2.3	4.82^e^	
	10	44444513	0.13	A/G	5.00 × 10^-8^	3.3	9.02^e^	*E2*
	13	37624457	0.36	T/C	3.13 × 10^-5^	2.3	3.79	17-1
	19	37391984	0.12	A/G	4.16 × 10^-6^	2.4	11.48^d^	
	19	47392861	0.37	T/C	2.75 × 10^-6^	2.5	0.69	5-10
Maturity date	7	8270118	0.30	A/G	4.61 × 10^-5^	1.9	2.90	2-1
	10	5392194	0.21	T/C	7.56 × 10^-6^	2.3	0.81	
	10	44753351	0.12	T/C	5.83 × 10^-19^	8.4	7.35^e^	*E2*
	18	59603446	0.06	A/G	5.9 × 10^-5^	1.9	0.19	29-8
	19	47390815	0.37	T/C	9.21 × 10^-13^	5.5	0.69	*E3*
Seed-coat color	3	42959913	0.21	G/A	6.97 × 10^-6^	5.0	1.25	
	5	34734860	0.36	C/T	3.06 × 10^-7^	7.5	8.28^e^	*CHS2*
	8	7589397	0.14	A/G	1.15 × 10^-16^	19.3	11.18^d^	*CHS*
	8	8462762	0.12	T/G	1.30 × 10^-19^	23.3	7.52^e^	*CHS*
	13	7681784	0.07	A/C	1.35 × 10^-5^	4.7	12.24^d^	
Pubescence color	3	47244893	0.18	A/G	3.46 × 10^-7^	8.3	0.00	
	6	17567713	0.20	G/A	4.54 × 10^-13^	18.9	0.39	
	6	18118558	0.40	T/C	1.08 × 10^-28^	48.2	0.00	*T*
	6	18583273	0.48	A/C	1.41 × 10^-25^	39.6	0.00	
Flower color	13	2833623	0.44	T/C	1.37 × 10^-11^	12.2	0.00	
	13	3301099	0.45	C/T	2.93 × 10^-29^	39.1	0.00	
	13	3657853	0.44	G/A	7.63 × 10^-35^	46.4	0.19	*W1*
	13	4198124	0.50	C/A	3.62 × 10^-28^	35.6	0.00	
	13	4559799	0.46	G/A	3.82 × 10^-40^	32.7	0.00	

^a^Chr., chromosome; ^b^Position in base pairs for the peak SNP according to soybean reference sequence of Williams 82; ^c^The significant SNP located in one of the QTL intervals as reported in other studies (www.soybase.org) . ^d^indicates a 1 % genome-wide cutoff level, ^e^indicates a 5 % genome-wide cutoff level

### GWAS for typical selection and non-selection-target traits

**Fig. 5:**
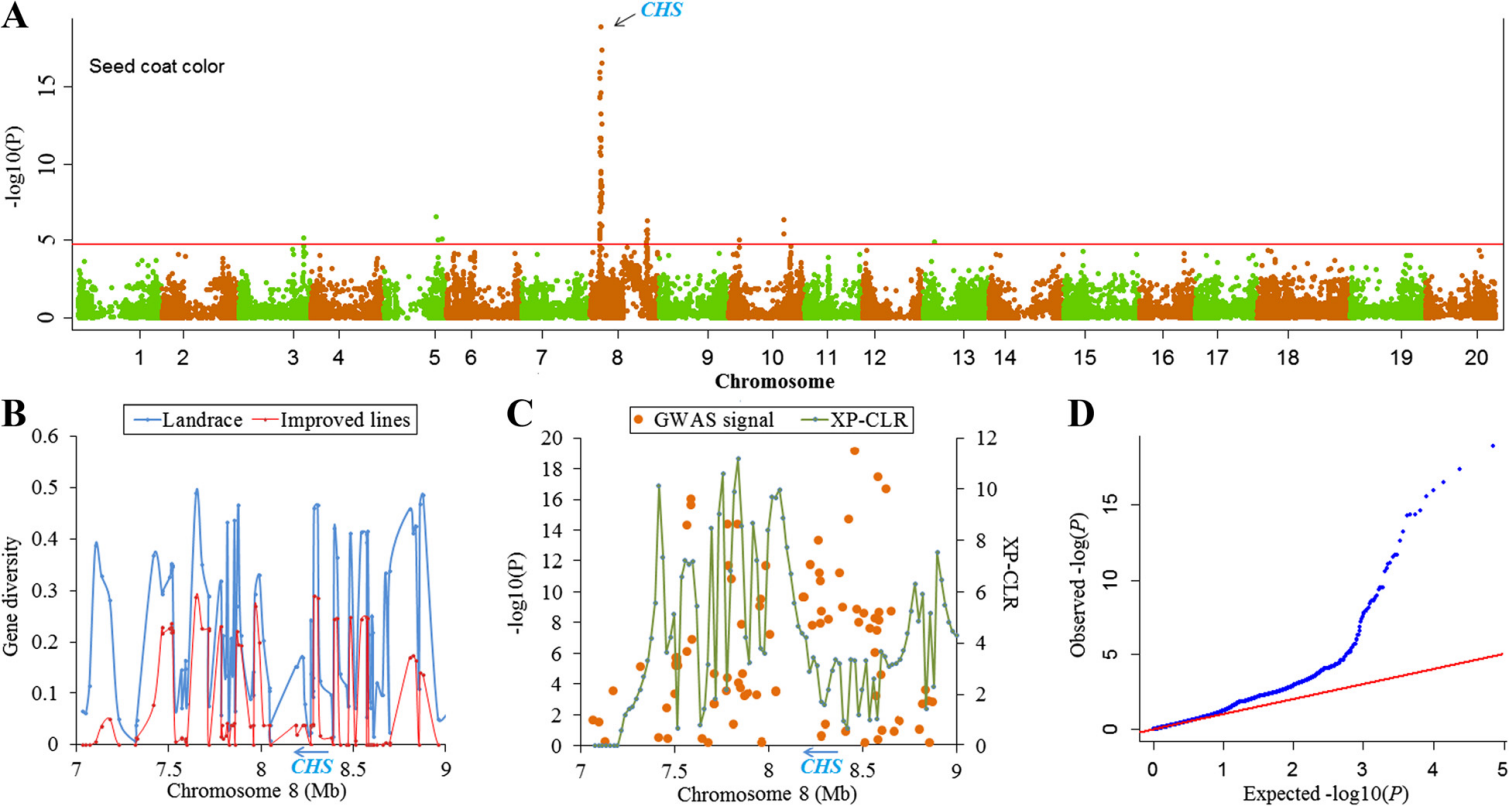
The visualization of the GWAS results and selection signals for selection trait (seed coat color). **a** Manhattan plots of MLM for seed coat color. The − log10 *P-*values from a genome-wide scan are plotted against the position on each of the 20 chromosomes. The horizontal red line indicates the genome-wide significance threshold (FDR < 0.05). **b** Gene diversity (*H*_*i*_) of genomic regions showing strong association signal on Chromosome 8. **c** XP-CLR and regional GWAS signals near *CHS*; gene orientation is indicated by the arrow. **d** Quantile-quantile (QQ) plot of MLM model for seed coat color

There is a high degree of variation in seed coat color within soybean landraces. However, yellow seed coat has been positively selected during soybean improvement. Thus seed coat color is a typical example of selection-target traits. A complex cluster of five chalcone synthase genes (*CHS1*, *CHS3*, *CHS4*, *CHS5*, and *CHS9*) on Chromosome 8, inhibiting coloration distribution, has been found to be associated with seed coat color [34, 35]. In our GWAS, we did detect a cluster of significant SNPs spanning a physical region of 1.7 Mb (7.3-9.0 Mb) around the five genes (Fig. 5a and c). The most significant SNP, Gm08-8462762 with *P*-value of 1.3 × 10^-19^, was located within the complex cluster of the 5 genes and explained 23 % of phenotypic variation (Fig. 5a). Note that both downstream and upstream of these genes showed strong selection signals with the highest XP-CLR value of 11.2 (Fig. 5c). Moreover, this genomic region showed obvious loss of genetic diversity (Fig. 5b).

**Fig. 6:**
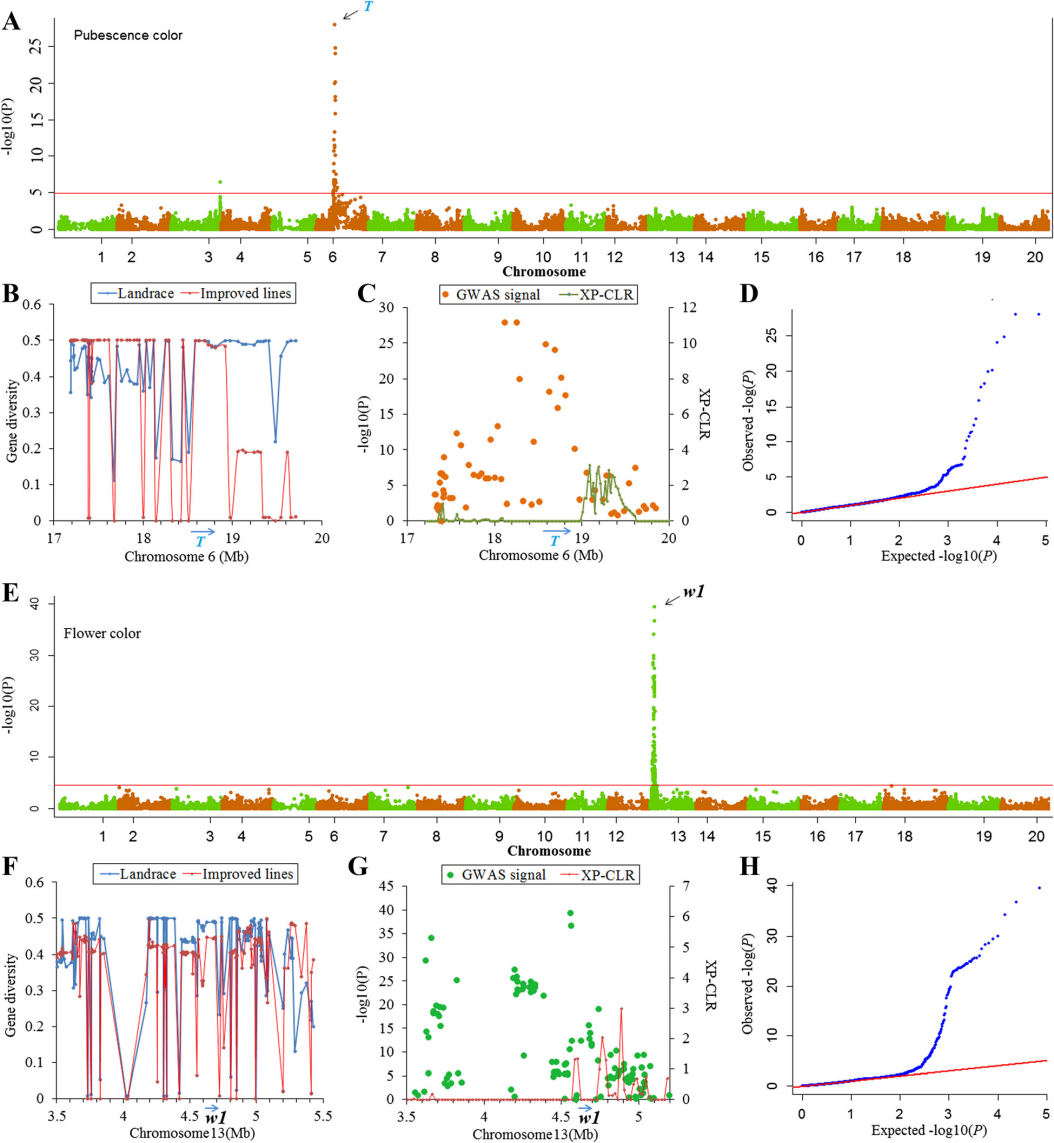
The visualization of the GWAS results and selection signals for two non-selection traits (pubescence and flower color). **a** Manhattan plots of MLM for pubescence color. The − log10 *P-*values from a genome-wide scan are plotted against the position on each of the 20 chromosomes. The horizontal red line indicates the genome-wide significance threshold (FDR < 0.05). **b** Gene diversity of genomic regions showing strong association signal on Chromosome 6. **c** XP-CLR and regional GWAS signals near *T* locus; gene orientation is indicated by the arrow. **d** Quantile-quantile (QQ) plot of MLM model for pubescence color. **e** Manhattan plots of MLM for flower color, as in **a**. **f** Gene diversity of genomic regions showing strong association signal on Chromosome 13. **g** XP-CLR and regional GWAS signals near *W1* locus; gene orientation is indicated by the arrow. **h** QQ plot of MLM for flower color

In contrast with seed coat color, there is no preferred color (tawny or gray) for pubescence in any soybean breeding program, so this trait can be taken as an example of a non-selection-target trait. A total of 46 SNPs were found to be significantly associated with pubescence color. Most of these SNPs, spanning a region of approximately 2 Mb (between 17-19 Mb on Chromosome 6), overlapped the *T* locus previously shown to control pubescence color [36] (Fig. 6c and Additional file 8). One of the significant SNPs, Gm06-18583273 with *P*-value of 1.4 × 10^-25^, lies ~50 kb upstream of *Glyma06g202300*, which encodes flavonoid 3’–hydroxylase. Previous research found that a single-base deletion in this gene would cause the pubescence color change from tawny to gray [37]. As expected, regions both downstream and upstream of this gene showed no significant selection signal with XP-CLR values ranging from 0 to 3.1 which did not exceed the cutoff value (Fig. 6c). Furthermore, this genomic region showed no obvious loss of genetic diversity (Fig. 6b). Similarly, there is no preferred color (purple or white) for flower color in most soybean breeding programs. A single region on Chromosome13 showed significant marker-trait associations (Fig. 6e and Additional file 8). The most significant SNP, Gm13-4559799 associated with flower color was found to be located just 2.2 kb downstream of *Glyma13g07210* (*W1* locus), a gene that codes for flavonoid 3’5’-hydroxylase [38]. As in the case of pubescence color, both downstream and upstream of this gene showed no selection signal with XP-CLR values ranging from 0 to 0.19, which did not exceed the cutoff value (Fig. 6g). Furthermore, this genomic region also showed no obvious loss of genetic diversity (Fig. 6f).

Taken together, these results indicate that, as expected, loci associated with selection-target traits have experienced much more positive selection than those associated with non-selection-target traits. Overall average XP-CLR values of loci associated with selection-target traits are about 20 times larger than those of non-selection-target traits (blue bar shown in Fig. 4). The results demonstrated that a genome-wide screen for selective sweeps can be used to identify loci of potential agronomic importance, even when the function and phenotype of those loci are unknown. Additionally, the accuracy of our GWAS approach was validated by the analysis performed for three simple Mendelian traits, for which the causal genes are known.

### GWAS for 6 quantitative traits

We further conducted GWAS for six quantitative traits including grain yield, plant height, lodging, maturity date, and protein and oil content. For grain yield, a total of 15 loci were identified in the elite cultivars and explained 36.6 % of the phenotypic variation. Since yield is a very complex trait and no candidate gene has been identified as a functional gene underlying soybean yield or yield component traits, it is difficult to assess the exhaustiveness and accuracy for these QTLs. However, when compared with chromosomal regions previously reported to contain yield QTLs (www.soybase.org) or GWAS signals, a total of 10 loci found in this study fall within such intervals or adjacent to previous GWAS signals (Additional files 8 and 9). For example, two significant SNPs, Gm01-55794390 and Gm20-41706616, detected in this work were adjacent to SNPs previously identified as being associated with number of pods per plant and seed weight, respectively [39]. Moreover, Gm20-41706616 (*P* = 1.06 × 10^-5^, R^2^ = 2.3 %) is adjacent to a cluster of genes that are homologous to *Arabidopsis APETALA2* genes (homeotic regulator) known to influence seed weight and size [40].

In the case of protein and oil content, a high negative correlation between these traits was found (Additional file 4). The seed protein content showed significant association with 9 loci located on 6 different chromosomes (Additional files 8 and 9). These loci explained 20.9 % of phenotypic variance in seed protein content. The oil content was significantly association with 10 loci located on 9 different chromosomes (Additional file 9); these loci explained 23.5 % of phenotypic variance. When compared with chromosomal regions previously reported to contain protein and oil QTLs (www.soybase.org), a total of 12 loci found in this study fall within such intervals or adjacent to previous GWAS signals (Additional file 8). For example, 3 significant SNPs, Gm07-7058915, Gm09-3379073 and Gm10-44274964, detected in this work fall within such intervals as well as adjacent to previously identified SNPs associated with protein content [12]. Moreover, four previously identified loci associated with oil content were also confirmed in this study (Table 3).

For lodging, height and maturity date, three highly correlated traits, two major regions were significantly associated with these traits, one on Chromosome 10 spanning from 44.3 to 44.9 Mb, and the other on Chromosome 19 spanning from 47.3 to 47.9 Mb (Additional file 9). The two regions coincide with the E2 and E3 maturity loci. Three SNPs (Gm10-44722784, Gm10-44723907 and Gm10-44724890) on chromosome 10 were strongly associated with maturity date, height and lodging simultaneously. These SNPs are located within an intron of *GmGIa*, a gene that has been demonstrated to be involved in soybean maturity and flowering time [41]. Similarly, a total of 11 SNPs on chromosome 19 spanning 47.3 to 47.9 Mb on Gm19 were strongly associated with maturity, as well as plant height (Additional files 8 and 9). These SNPs are adjacent to *GmPhyA3* gene, a phytochrome receptor corresponding to the *E3* maturity locus [42]. The co-localization of significant regions for these traits in the current work is similar with that reported by Sonah et al. [39]. This could be the result of pleiotropy or closely linked genes within the same region [43].

Although we found that average XP-CLR values of regions associated with selection-target traits are about 20 times larger than those of non-selection-target traits, one third of the regions associated with selection-target traits showed no significant signals (<5 % genome-wide cutoff level) of selective sweeps (Table 3). Failure to detect the significant signal may be attributed to two reasons. First, since the XP-CLR method relies on multi-locus allele frequency differentiation between two populations [13], lack of polymorphic SNP markers in the specific region among our two populations may lead to failure to detect a significant signal. Second, selection is likely to have affected standing variation. If the selected mutations were present in different haplotypes before selection was initiated, the XP-CLR statistic may have limited power to detect selection [14].

## Conclusions

Our results showed that modern breeding has introduced detectable genetic changes to the soybean genome. A genome-wide screen for artificial selection identified 125 genomic regions of potential agronomic importance. By means of association mapping, a set of new loci as well as refined map locations of known loci were found to contribute to the phenotypic variance of 9 agronomic traits, which will be attractive candidates for further investigation. Major genomic regions, associated with selection traits, overlap with candidate selection region, whereas no overlap of this kind occurred for the non-selection traits. This indicates the potential for using comparative genetic techniques to identify genomic regions relating to phenotypes of importance to soybean breeders. Ultimately, uncovering the genetic architecture of agronomic traits will provide the basis for improving yield, quality and sustainability of soybean.

## Methods

### Sampling and genotyping

The plant materials included genotypes from two soybean populations. The first population consisted of 342 traditional landraces from multiple geographic origins including China, Japan, Korea, Kyrgyzstan and Russia. The soybean landrace was defined as a locally adapted, traditional variety of a domesticated soybean that has developed over time, through adaptation to its natural and cultural environment. There are 21 accessions belong to 80 ancestral soybean lines listed by Gizlice et al. [5]. To maximize the diversity sampled, these landraces were selected based on representative variations, detected by SoySNP50K BeadChip [17], among all soybean landraces from maturity groups I, II and III. The second population consisted of 1062 improved lines released from 2007 to 2012, which were chosen to represent a range of materials developed for the U.S., North Central production area. Based on the kinship analysis described below, less than 7 % of accessions have close familial relatedness (Additional file 10). Further information for each accession (commercial name, origin and subpopulation association) is given in Additional file 11.

Soybean genomic DNA was extracted from young leaf tissue following the standard CTAB method [44]. All the accessions were genotyped using the Illumina SoySNP50k iSelect BeadChip (Illumina, San Diego, USA) which consists of 52,041 SNPs [17]. Genotypes were called using the program GenomeStudio (Illumina, San Diego, USA). The quality of each SNP was checked manually as previously reported [45]. SNPs without physical position information and with low quality (call rate < 80 %, minor allele frequency < 0.05) across all samples were removed from the dataset.

### Population genetic analyses

Summary statistics were computed for the polymorphic SNP data sets in both landraces and improveed lines. The statistics, including the number of alleles (*A*_*i*_) and gene diversity index (*H*_*i,*_, ref. [46]), were calculated by Powermarker 3.25 [47]. Principal component analysis and Neighbor-joining trees were applied to infer population stratification. A pairwise distance matrix derived from the Nei’s genetic distance for all polymorphic SNPs was calculated to construct Neighbor-joining trees using PowerMarker 3.25. Principal component analysis was done using EIGENSTRAT [48] based on 15,908 and 9578 SNPs with minor allele frequency (MAF) >20 % and physical distance >60 kb for improved lines and landraces, respectively. Kinship matrixes (*K*) were calculated using TASSEL4.0 [49] to determine relatedness among individuals based on the same sets of SNPs for the two populations (Additional file 10). Linkage disequilibrium parameter (*r*^2^) for estimating the degree of LD between pair-wise SNPs was calculated using the software TASSEL4.0. The extent of LD decay was measured as the chromosomal distance at which the average pairwise correlation coefficient (*r*^*2*^) dropped to half its maximum value.

Evidence for selection across the genome during improvement was evaluated between improved lines and landraces. A cross-population composite likelihood ratio test (XP-CLR) was used to perform the genome scan for selection [13]. A 0.05-cM sliding window with 20 kb steps across the whole genome scan was used. To ensure comparability of the composite likelihood score in each window, the number of SNPs assayed in each window was fixed to 50, and pairs of SNPs in high LD (*r*^*2*^ > 0.75) were down-weighted to minimize the effect of dependence on the composite likelihood score. Likelihood ratio (XP-CLR) was estimated and assigned to each 20-kb window [13]. We determined empirical cutoffs for the top 1 % of signals genome-wide and considered these strongest signals to indicate candidate selection regions. To account for the non-independence of XP-CLR scores along the physical map, regions within the same LD block were grouped and considered as putatively selected features. The LD block was identified using the default algorithm implemented in the *define blocks* function of Haploview 4.2 software [50].

### Phenotyping

For each soybean landrace accession, pure line seeds of all accessions were obtained from the U.S. Department of Agriculture Soybean Germplasm Collection (U.S. Department of Agriculture, Agriculture Research Station, University of Illinois, Urbana, IL). The landrace accessions were planted in single row plots, 6 m long with 0.75 m row spacing, at the Agronomy Farm of Michigan State University. Three Mendelian traits, flower color, seed coat color and pubescence color were investigated. Measurement methodology of each trait was described in Additional file 12. All improved soybean lines were evaluated in the fields in Allegan, Hillsdale, Ingham, Saint Joseph, Lenawee, Saginaw and Sanilac counties, Michigan during the growing season (May – October) from 2007 to 2012. Seed was planted in 6-row plots, 6 m long with 0.38 m row spacing, at a depth of 3.8 cm. Planting rate was 72,900 seeds per hectare. At each location, varieties were replicated four times in a lattice design. The plots were trimmed to a length of 4.3 m and the center four rows were harvested for yield estimation. Six agronomic traits, grain yield, maturity date, plant height, lodging, protein and oil content, were investigated. Measurement methodology of each trait is described in Additional file 12. Other detailed information on the performance trial and all phenotypic data are available from the following website http://www.css.msu.edu/varietytrials/soybean/Soybean_Home_Page.htm. Since the improved lines were phenotyped in multiple environments, the best linear unbiased predictors (BLUPs) were used for the overall association analysis of the soybean improved lines. Analysis of variance (ANOVA) for the phenotypic data was performed with the R package, lm(stats) and anova.lm(stats).

### Genome-wide association analysis

Two different models, general linear model (GLM) and mixed linear model (MLM), were used to test the associations between the SNPs (MAF > 5 %) and phenotypic variations. The GLM and MLM can be expressed as *y* = *Xα* + *e* and *y* = *Xα* + *Pβ + Kμ + e*, respectively, where *y* is the vector of phenotypic observations, *α is* the vector of SNP effects; *β* is the vector of population structure effects; *μ* is the vector of kinship background effects; *e* is the vector of residual effects; *P* is the PCA matrix relating *y* to *β*; *X* and *K* are incidence matrices of 1 s and 0 s relating *y* to *α* and *μ*, respectively [51]. The top six principal components were used to build up the *P* matrix for population structure correction in the two panels. Analyses were performed by the software TASSEL 4.0, which implemented the EMMA and P3D algorithms to reduce computing time [52]. False discovery rate (FDR) ≤ 0.05 was used to identify significant associations. Additional file 13 is a flow chart showing the overall experimental design.

### Availability of supporting data

The data sets supporting the results of this article are included within this article and its additional files.

## Additional files

Additional file 1:
**Population-differentiation statistics (** ***F***_**ST**_ **) among subpopulation in soybean landraces and improved lines.** (DOCX 17 kb) Additional file 2:
**Distribution of accessions in each subgroup based on genetic distance in landraces and improved lines.** (DOCX 19 kb) Additional file 3:
**Detail Information of 125 selection features and corresponding candidate genes.** (XLSX 37 kb) Additional file 4:
**The phenotypic variation and correlation analysis for 6 quantitative traits.** (DOCX 16 kb) Additional file 5:
**The frequency distribution of variation of 9 traits in tested soybean accessions.** (DOCX 87 kb) Additional file 6:
**Quantile-quantile (QQ) plots of general linear model (GLM) for 9 agronomic traits.** (DOCX 232 kb) Additional file 7:
**Quantile-quantile (QQ) plots of mixed linear model (MLM) for 9 agronomic traits.** (DOCX 90 kb) Additional file 8:
**The descriptive summary of GWAS for 9 agronomic traits in soybean.** (XLSX 44 kb) Additional file 9:
**Is a figure showing the visualization of the GWAS results for 6 quantitative traits.** The − log10 P-values from a genome-wide scan are plotted against the position on each of the 20 chromosomes. The horizontal red line indicates the genome-wide significance threshold (FDR <0.05). (DOCX 497 kb) Additional file 10:
**The heat map showing kinship value between individual accessions among the landraces and the improved lines. Pairwise kinship values are shown as color-index heat map.** (DOCX 963 kb) Additional file 11:
**The list of 342 soybean landraces and 1062 improved lines sampled in this study.** (XLSX 78 kb) Additional file 12:
**The list of phenotypes used in this study and summary of tested environments.** (DOCX 19 kb) Additional file 13:
**Flow chart of overall experimental design.** (PPTX 70 kb)

## Abbreviations

XP-CLR: Cross-population composite likelihood ratio
GWAS: Genome-wide association
SNPs: Single nucleotide polymorphisms
LD: Linkage disequilibrium
QTL: Quantitative trait locus
MAF: Minor allele frequency
NJ: Neighbor-joining
PCA: Principal components analysis
QQ: Quantile-quantile
GLM: General linear model
MLM: Mixed linear model
