# Supplementary material for: Genomic consequences of selection and genome-wide association mapping in soybean
Source: BMC Genomics. 2015 Sep 3;16(1):671. doi: 10.1186/s12864-015-1872-y (PMC4559069; doi:10.1186/s12864-015-1872-y)
Supplement: Additional file 1: — Population-differentiation statistics ( F ST ) among subpopulation in soybean landraces and improved lines. (DOCX 17 kb) [file 12864_2015_1872_MOESM1_ESM.docx]

|  | Landraces | | | | |  |  |  |  | Improved lines | | | | | | |  |
| --- | --- | --- | --- | --- | --- | --- | --- | --- | --- | --- | --- | --- | --- | --- | --- | --- | --- |
|  | 1 | 2 | 3 | 4 | 5 | 6 |  |  |  | 1 | 2 | 3 | 4 | 5 | 6 | 7 | 8 |
| 1 | 0 |  |  |  |  |  |  |  | 1 | 0 |  |  |  |  |  |  |  |
| 2 | 0.09376 | 0 |  |  |  |  |  |  | 2 | 0.0659 | 0 |  |  |  |  |  |  |
| 3 | 0.08929 | 0.12154 | 0 |  |  |  |  |  | 3 | 0.11644 | 0.14896 | 0 |  |  |  |  |  |
| 4 | 0.13637 | 0.07491 | 0.15545 | 0 |  |  |  |  | 4 | 0.12813 | 0.16686 | 0.18171 | 0 |  |  |  |  |
| 5 | 0.13126 | 0.06523 | 0.11533 | 0.08368 | 0 |  |  |  | 5 | 0.08924 | 0.11969 | 0.15021 | 0.13038 | 0 |  |  |  |
| 6 | 0.08256 | 0.08959 | 0.10869 | 0.1158 | 0.07724 | 0 |  |  | 6 | 0.11311 | 0.11766 | 0.15458 | 0.15001 | 0.10829 | 0 |  |  |
|  |  |  |  |  |  |  |  |  | 7 | 0.16418 | 0.19686 | 0.25725 | 0.19183 | 0.19561 | 0.17772 | 0 |  |
|  |  |  |  |  |  |  |  |  | 8 | 0.10262 | 0.1259 | 0.14615 | 0.07421 | 0.10146 | 0.0862 | 0.15214 | 0 |

**Additional file 1 The population-differentiation statistics (*F*_ST_) among subpopulation in soybean landraces and improved lines.**
