# Supplementary material for: Genomic consequences of selection and genome-wide association mapping in soybean
Source: BMC Genomics. 2015 Sep 3;16(1):671. doi: 10.1186/s12864-015-1872-y (PMC4559069; doi:10.1186/s12864-015-1872-y)
Supplement: Additional file 2: — Distribution of accessions in each subgroup based on genetic distance in landraces and improved lines. (DOCX 19 kb) [file 12864_2015_1872_MOESM2_ESM.docx]

**Additional file 2 Distribution of accessions in each subgroup based on genetic distance in landraces and improved lines**

| Population | |  | | Clusters of NJ tree | | | | | | | | | | | | | | | | |  |  |
| --- | --- | --- | --- | --- | --- | --- | --- | --- | --- | --- | --- | --- | --- | --- | --- | --- | --- | --- | --- | --- | --- | --- |
| Landraces |  | | 1 | | | 2 | | 3 | | 4 | | 5 | | 6 | |  | | | |  |  |  |
|  | NC | | 25 | | | 29 | | 5 | | 32 | | 45 | | 4 | |  | | | |  |  |  |
|  | HHC | | 47 | | | 1 | | 0 | | 6 | | 3 | | 0 | |  | | | |  |  |  |
|  | SC | | 7 | | | 1 | | 0 | | 0 | | 0 | | 0 | |  | | | |  |  |  |
|  | Japan | | 2 | | | 21 | | 5 | | 2 | | 1 | | 1 | |  | | | |  |  |  |
|  | Korea | | 2 | | | 20 | | 0 | | 5 | | 0 | | 0 | |  | | | |  |  |  |
|  | Far East | | 4 | | | 8 | | 1 | | 5 | | 10 | | 2 | |  | | | |  |  |  |
|  | other | | 14 | | | 3 | | 0 | | 9 | | 15 | | 7 | |  | | | |  |  |  |
| Improved lines | *χ*^2^ test | | *χ*^2^= 228.56 >*χ*^2^_0.01,30_ = 50.89, *P*<0.001 | | | | | | | | | | | | |  | | | |  |  |  |
|  |  | | Clusters of NJ tree | | | | | | | | | | | | | | | | | |  |  |
|  |  |  | 1 | | 2 | | 3 | | 4 | | 5 | | 6 | | 7 | | 8 | | | |  |  |
|  | MG I  MG II  MG III | | 14 | | 82 | | 3 | | 57 | | 5 | | 26 | | 0 | | | 24 | | |  |  |
|  |  |  | 60 | | 150 | | 11 | | 206 | | 19 | | 75 | | 7 | | | 94 | | |  |  |
|  |  |  | 34 | | 50 | | 1 | | 62 | | 8 | | 24 | | 7 | | | 43 | | |  |  |
|  | *χ*^2^ test | | *χ*^2^= 41.32> *χ*^2^_0.01,14_ = 29.14, *P*<0.001 | | | | | | | | | | | | | | | |  | |  |  |

NC: North China, HHC: Huanghuai China, SC:South China, MG, maturity group
