# Supplementary material for: Genomic consequences of selection and genome-wide association mapping in soybean
Source: BMC Genomics. 2015 Sep 3;16(1):671. doi: 10.1186/s12864-015-1872-y (PMC4559069; doi:10.1186/s12864-015-1872-y)
Supplement: Additional file 4: — The phenotypic variation and correlation analysis for 6 quantitative traits. (DOCX 16 kb) [file 12864_2015_1872_MOESM4_ESM.docx]

**Additional file 4 Phenotypic variation and correlation analysis for 6 quantitative traits.**

|  |  | Grain Yield (kg/hec.) | Protein content (%) | Oil content (%) | Lodging | Plant Height (cm) | Maturity date |
| --- | --- | --- | --- | --- | --- | --- | --- |
| Descriptive | Range | 2390.6–5400.4 | 37.7-48.8 | 17.5–22.9 | 1.0-3.5 | 52.5-121.4 | 109-140 |
| statistics | Mean ± s.d^1^. | 3824 ± 456.1 | 42.5±1.5 | 20.5±0.8 | 1.7±0.5 | 88.3±10.8 | 125±5.6 |
| ANOVA | G^2^ | ** | ** | ** | ** | ** | ** |
|  | G×E^3^ | ** | ** | ** | ** | ** | ** |
| Correlation | Grain Yield | 1 |  |  |  |  |  |
| coefficient c | Protein content | -0.23* | 1 |  |  |  |  |
|  | Oil content | 0.28 | -0.74** | 1 |  |  |  |
|  | Lodging | -0.20** | 0.13 | -0.3 | 1 |  |  |
|  | Plant Height | 0.32** | -0.02 | -0.08* | 0.45** | 1 |  |
|  | Maturity date | 0.22* | -0.02 | -0.21* | 0.48** | 0.54** | 1 |

^1^s.d., standard deviation; ^2^G, Genotype across different environments; ^3^ G×E , Genotype ×environment(, including genotype× year , genotype× location, genotype × year × location); *Significant at *P* < 0.05; **Significant at *P* < 0.01
