# Supplementary figures and images for: Genomic consequences of selection and genome-wide association mapping in soybean
Source: BMC Genomics. 2015 Sep 3;16(1):671. doi: 10.1186/s12864-015-1872-y (PMC4559069; doi:10.1186/s12864-015-1872-y)

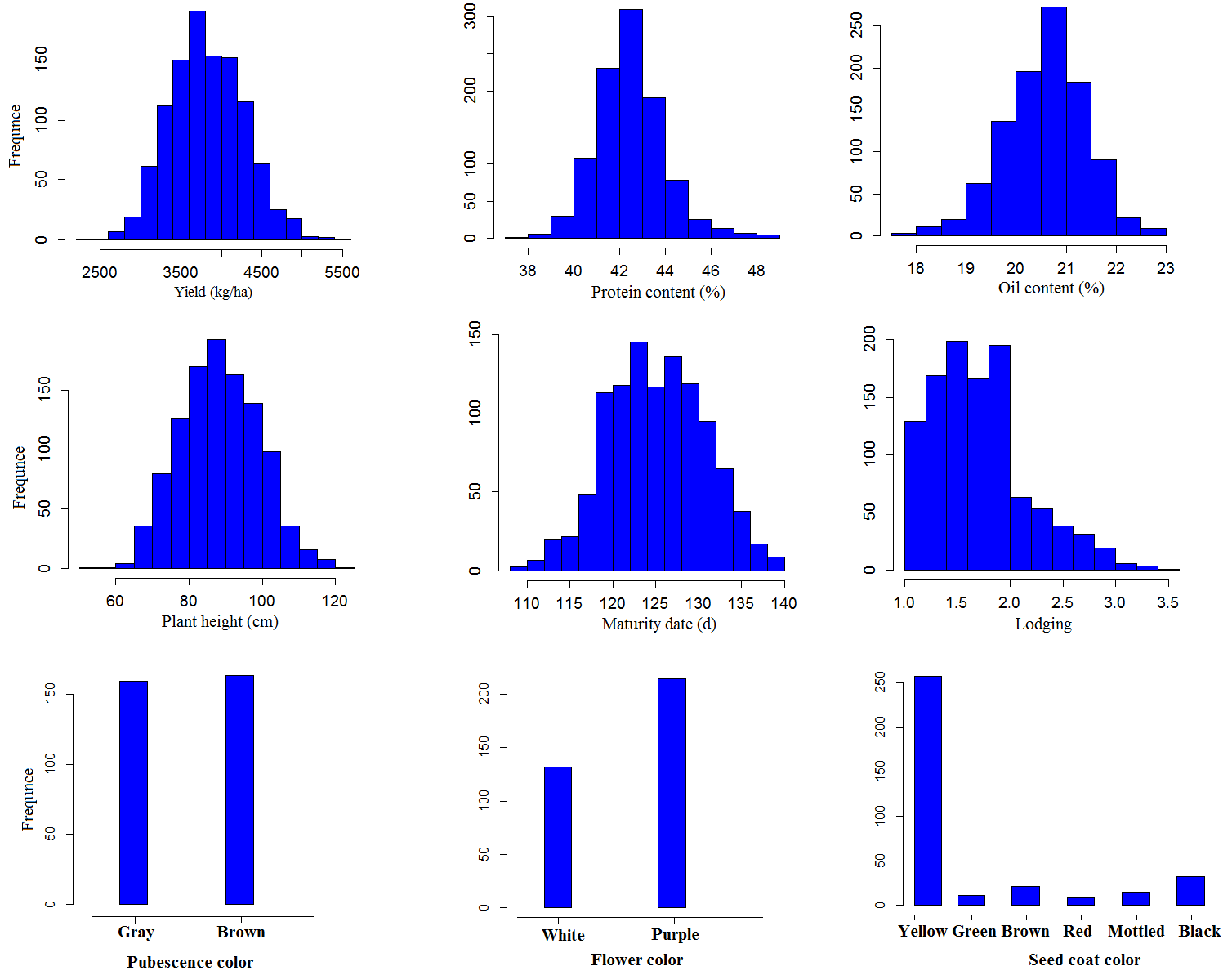
**Additional file 5 Frequency distribution of variation of 9 traits in tested soybean accessions.**

Supplement: Additional file 5: — The frequency distribution of variation of 9 traits in tested soybean accessions. (DOCX 87 kb) [file 12864_2015_1872_MOESM5_ESM.docx]

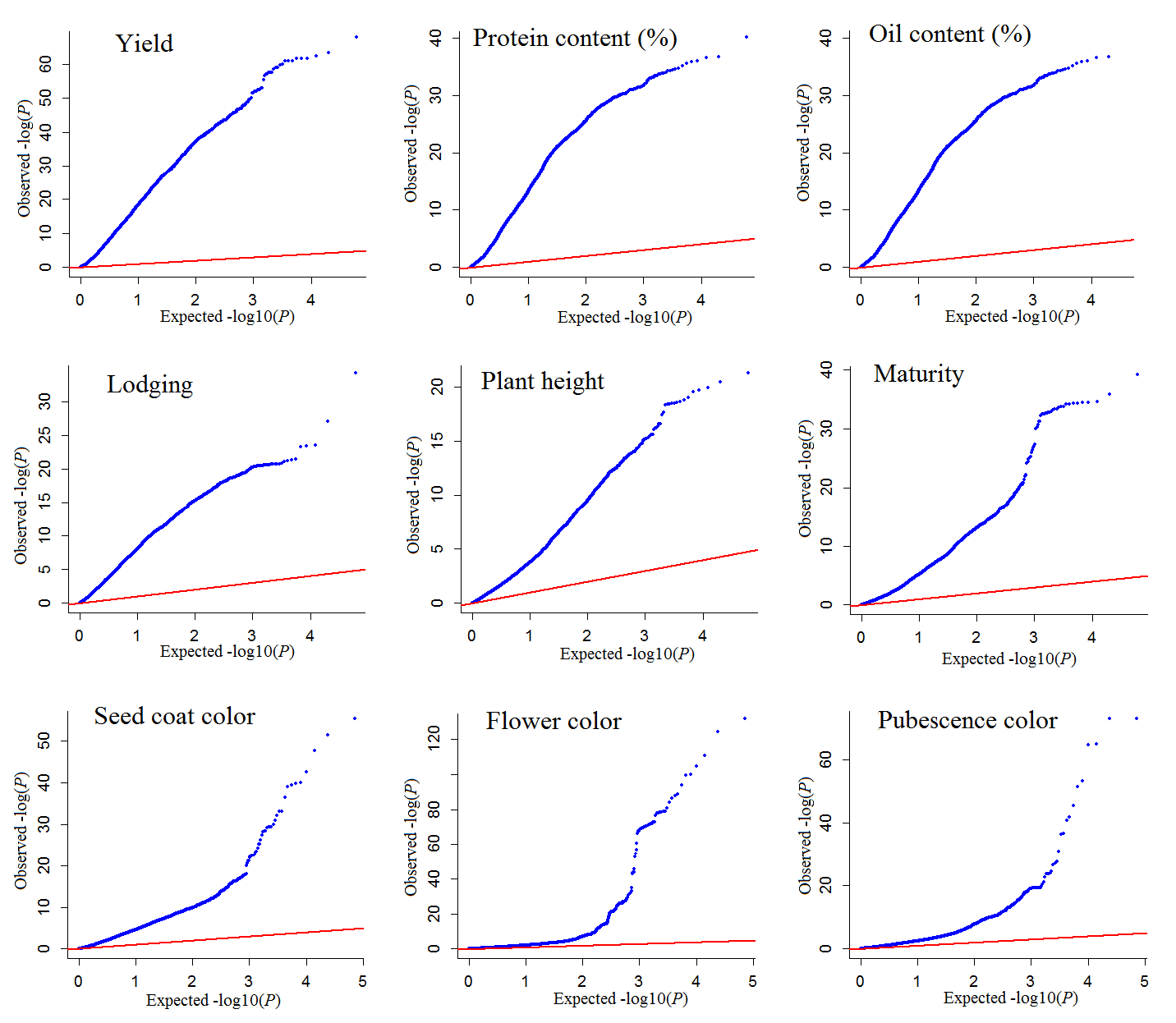


**Additional file 6 Quantile-quantile (QQ) plot of general linear model (GLM) for 9 agronomic traits.**

Supplement: Additional file 6: — Quantile-quantile (QQ) plots of general linear model (GLM) for 9 agronomic traits. (DOCX 232 kb) [file 12864_2015_1872_MOESM6_ESM.docx]

**
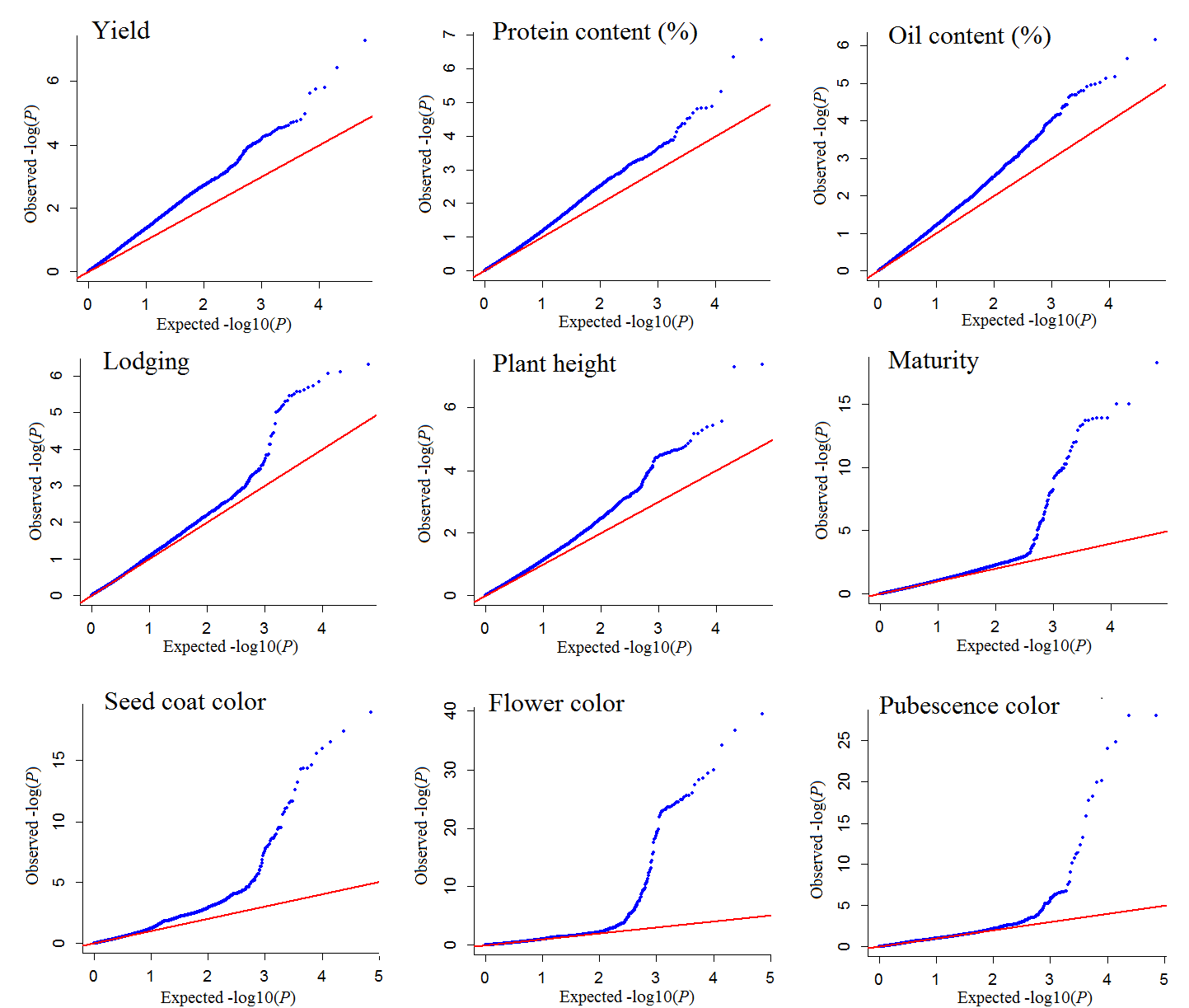
 Additional file 7 Quantile-quantile (QQ) plot of mixed linear model (MLM) for 9 agronomic traits.**

Supplement: Additional file 7: — Quantile-quantile (QQ) plots of mixed linear model (MLM) for 9 agronomic traits. (DOCX 90 kb) [file 12864_2015_1872_MOESM7_ESM.docx]
