# Supplementary material for: Genomic consequences of selection and genome-wide association mapping in soybean
Source: BMC Genomics. 2015 Sep 3;16(1):671. doi: 10.1186/s12864-015-1872-y (PMC4559069; doi:10.1186/s12864-015-1872-y)
Supplement: Additional file 9: — Is a figure showing the visualization of the GWAS results for 6 quantitative traits. The − log10 P-values from a genome-wide scan are plotted against the position on each of the 20 chromosomes. The horizontal red line indicates the genome-wide significance threshold (FDR <0.05). (DOCX 497 kb) [file 12864_2015_1872_MOESM9_ESM.docx]

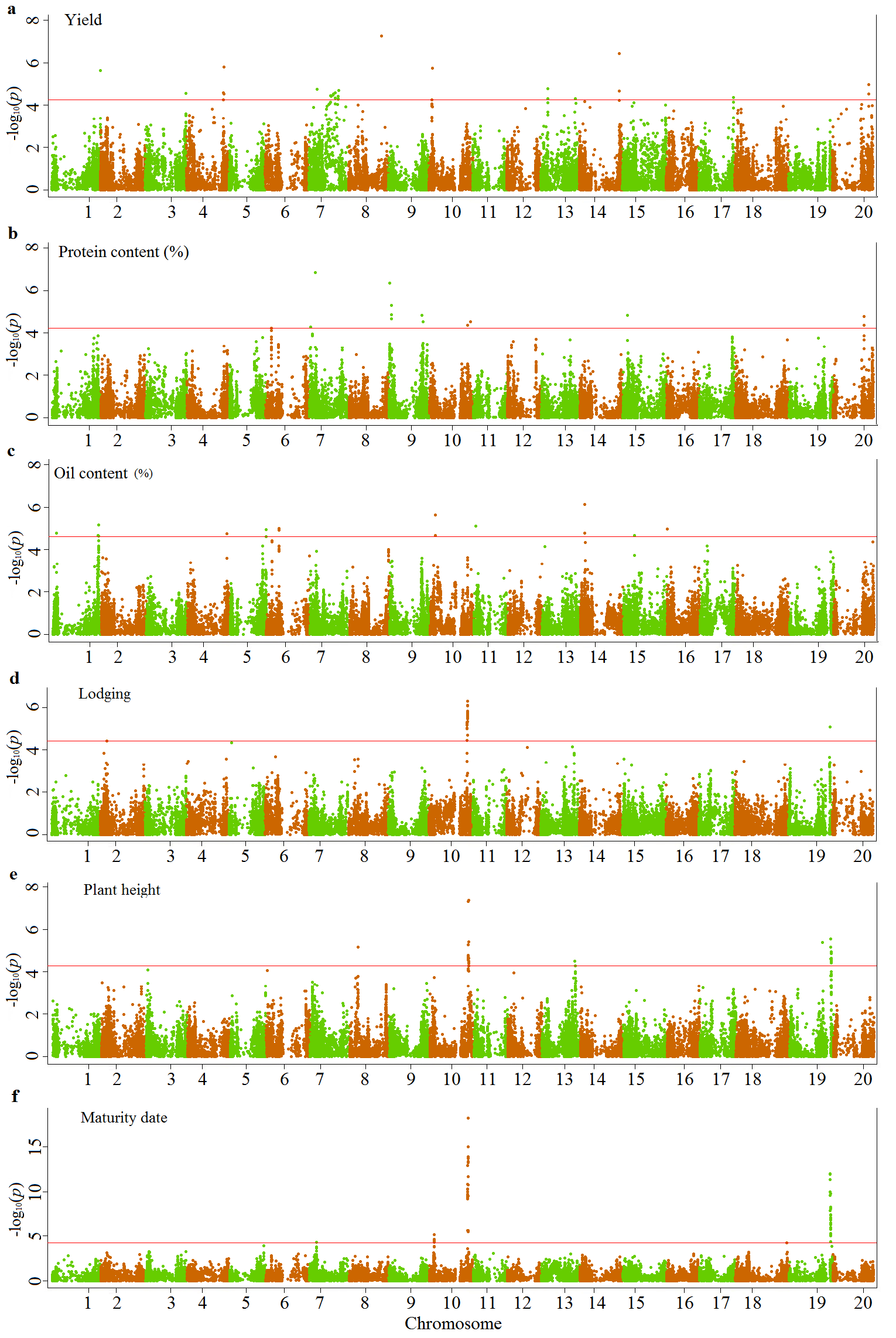


**Additional file 9 The visualization of the GWA mapping results for 6 quantitative traits.**

The −log10 *P-*values from a genome-wide scan are plotted against the position on each of the 20 chromosomes. The horizontal red line indicates the genome-wide significance threshold (FDR<0.05).
