# Supplementary material for: Genomic consequences of selection and genome-wide association mapping in soybean
Source: BMC Genomics. 2015 Sep 3;16(1):671. doi: 10.1186/s12864-015-1872-y (PMC4559069; doi:10.1186/s12864-015-1872-y)
Supplement: Additional file 10: — The heat map showing kinship value between individual accessions among the landraces and the improved lines. Pairwise kinship values are shown as color-index heat map. (DOCX 963 kb) [file 12864_2015_1872_MOESM10_ESM.docx]

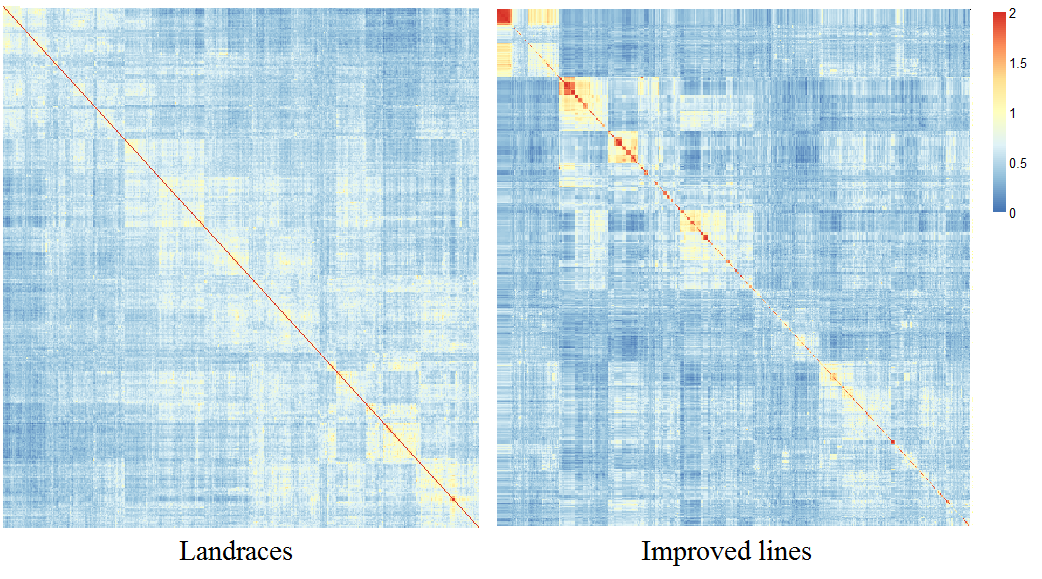


**Additional file 10 Heat map showing kinship value between individual accessions among landraces and improved lines**. Pairwise kinship values are shown as color-index heat map.
