# Supplementary material for: Genomic consequences of selection and genome-wide association mapping in soybean
Source: BMC Genomics. 2015 Sep 3;16(1):671. doi: 10.1186/s12864-015-1872-y (PMC4559069; doi:10.1186/s12864-015-1872-y)
Supplement: Additional file 12: — The list of phenotypes used in this study and summary of tested environments. (DOCX 19 kb) [file 12864_2015_1872_MOESM12_ESM.docx]

**Additional file 12 List of phenotypes used in this study and summary of tested environments.**

| **Trait category** | **Trait name** | **Trait description or rating scale** | **Test Environments** | |
| --- | --- | --- | --- | --- |
|  |  |  | **Year** | **Location** |
| Selection-target trait | Grain yield | Yield is expressed as kg per hectare at 13% moisture basis calculated form yield of center four rows of 6 rows plots (6 m long with 0.38 m row spacing) | 2007 to 2012 | 7^a^ |
|  | Protein content | Protein content of the seed was determined using near-infrared reflectance and is expressed on a 13% moisture basis | 2007 to 2012 | 3^b^ |
|  | Oil content | Oil content of the seed was determined using near-infrared reflectance and is expressed on a 13% moisture basis | 2007 to 2012 | 3 |
|  | Plant height | Plant height was measured at maturity from the soil surface to the tip of the main stem | 2007 to 2012 | 7 |
|  | Maturity data | Entries were considered mature when 95% of the pods had attained their final color and would crack under finger pressure | 2007 to 2012 | 7 |
|  | Lodging | Lodging scores reflect the erectness of the plants before harvest. Ratings are based on the following scale: 1= Almost all plants erect; 2= All plants leaning slightly, or fewer than 25% of the plants down; 3= All plants leaning moderately (45%), or 25% to 50% of the plants down; 4= All plants leaning considerably, or 50% to 80% of the plants down; 5= Almost all plants down. | 2007 to 2012 | 7 |
|  | Seed coat color | Color of soybean mature seed. Rating are based on the following scale:  1= Yellow; 2= Green;3= Brown;4= Dark red;5= Dappled color; 6= Black | 2013 | 1^c^ |
| Non-selection-target trait | Pubescence color | Color of pubescence on mature pod. Two colors, tawny or gray, were recorded, than transformed to binary numerical data (1 and 2) for data analysis. | 2013 | 1 |
|  | Flower color | Two colors, white and purple, were recorded, than transformed to binary numerical data (1 and 2) for data analysis. | 2013 | 1 |

a,7 location including Allegan, Hillsdale, Ingham, Saint Joseph, Lenawee, Saginaw and Sanilac in Michigan.

b, 3 location including Ingham, Saginaw and Lenawee in Michigan

c, the only one location is Ingham in Michigan
