# Supplementary material for: Genomic consequences of selection and genome-wide association mapping in soybean
Source: BMC Genomics. 2015 Sep 3;16(1):671. doi: 10.1186/s12864-015-1872-y (PMC4559069; doi:10.1186/s12864-015-1872-y)
Supplement: Additional file 13: — Flow chart of overall experimental design. (PPTX 70 kb) [file 12864_2015_1872_MOESM13_ESM.pptx]

## Slide 1
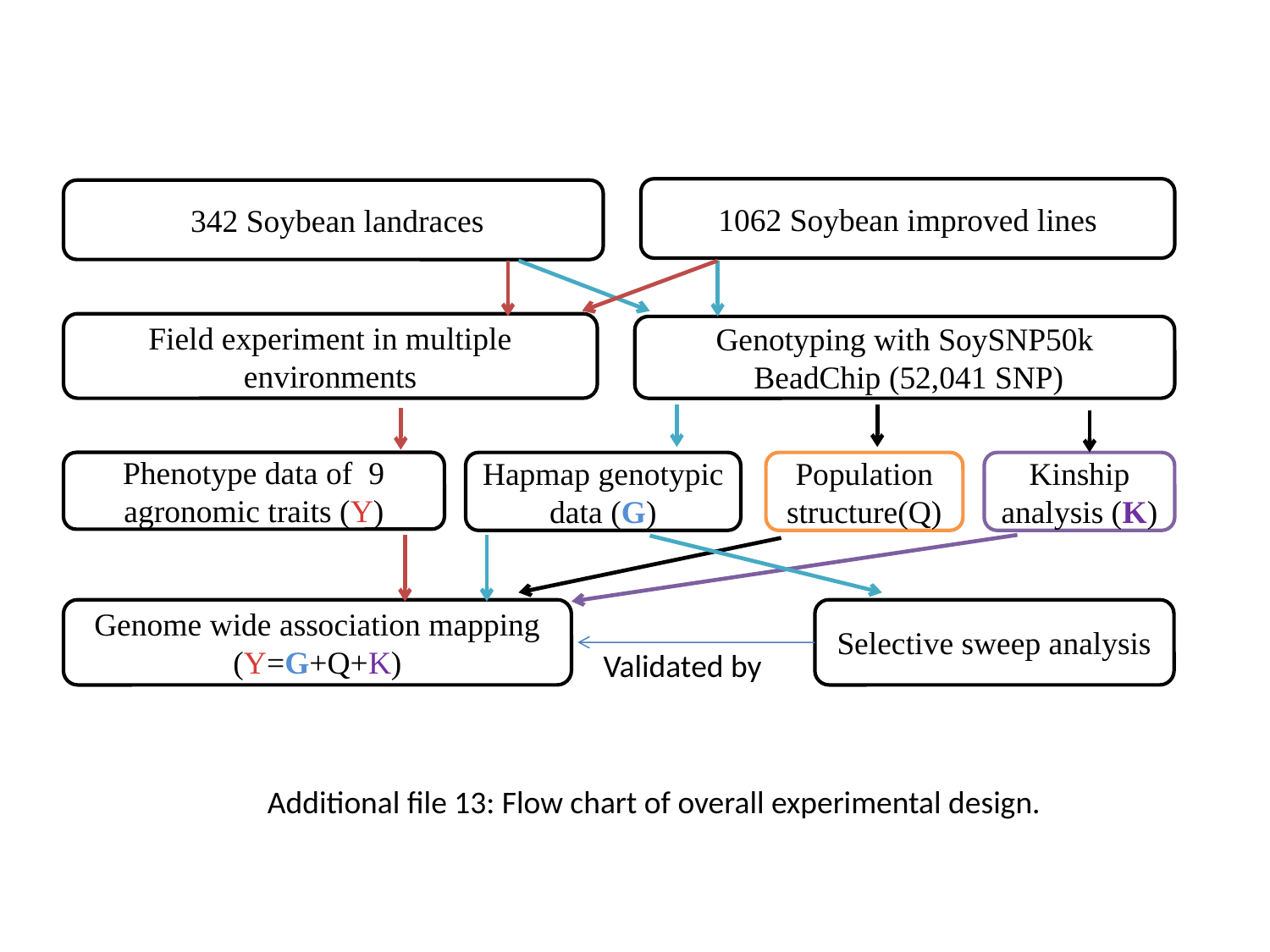

342 Soybean landraces
1062 Soybean improved lines
Field experiment in multiple environments
Genotyping with SoySNP50k
 BeadChip (52,041 SNP)
Phenotype data of 9 agronomic traits (Y)
Hapmap genotypic data (G)
Population structure(Q)
Kinship analysis (K)
Genome wide association mapping
(Y=G+Q+K)
Selective sweep analysis
Validated by
Additional file 13: Flow chart of overall experimental design.
